# Experience, Process, and Impact of Involving Informal Caregivers of People With Dementia as Public Contributors to Inform the Development of a Complex Intervention: A Mixed‐Methods Study

**DOI:** 10.1111/hex.70382

**Published:** 2025-08-17

**Authors:** Frida Svedin, Ida Österman Menander, Oscar Blomberg, Anders Brantnell, Paul Farrand, Theresia Lückner, Kristina Sundelin, Joan Turney, Anna Cristina Åberg, Joanne Woodford

**Affiliations:** ^1^ CIRCLE—Complex Intervention Research in Health and Care, Department of Women's and Children's Health Uppsala University Uppsala Sweden; ^2^ Industrial Engineering and Management, Department of Civil and Industrial Engineering Uppsala University Uppsala Sweden; ^3^ Clinical Education, Development and Research (CEDAR); Psychology University of Exeter Devon UK; ^4^ INVOLVERA Public Advisory Group, CIRCLE—Complex Intervention Research in Health and Care, Department of Women's and Children's Health Uppsala University Uppsala Sweden; ^5^ Clinical Geriatrics, Department of Public Health and Caring Sciences Uppsala University Uppsala Sweden; ^6^ Medical Sciences, School of Health and Welfare Dalarna University Falun Sweden

**Keywords:** codesign, dementia research, evaluation, intervention development, patient and public involvement

## Abstract

**Introduction:**

Public contribution is increasingly prioritised by research institutions, funding bodies, and policymakers globally. However, the evidence base for the impact of public contribution remains limited. Researchers and public contributors' experiences of such activities are also rarely reported. We worked alongside a Public Advisory Group (PAG) consisting of informal caregivers of people with dementia during a series of studies to inform the development and adaptation of a guided low‐intensity behavioural activation intervention for people with dementia (INVOLVERA). The overall aim of the current study was to explore the experience, process, and impact of involving caregivers of people with dementia as public contributors during the intervention development phase of INVOLVERA.

**Methods:**

Public contribution activities were recorded using impact logs from PAG meetings, from which public contributors' suggestions for the intervention were extracted and categorised, and the implementation rate calculated. Semi‐structured interviews were conducted with public contributors (*n* = 4) and researchers (*n* = 3), and analysed using manifest content analysis.

**Results:**

Public contributors made 158 suggestions across nine PAG meetings, with 76% of these implemented by the researchers. Analysis of interviews generated three categories: *Perceived impacts, Interactions and facilitators*, and *Future challenges and opportunities*. Interviews suggested public contribution activities positively impacted the research (e.g., improving intervention acceptability) and those involved (e.g., new knowledge and skills). Public contributors provided valuable recommendations for involving people with dementia and male caregivers in future activities.

**Conclusions:**

Findings illustrate a positive impact of public contribution activities on the research and those involved. This underscores the important role of public contribution during the development of complex interventions and further emphasises the need for comprehensive reporting on the impact of such activities. We believe this study strengthens the evidence base for public contribution and offers practical insights into fostering effective partnerships with public contributors.

**Public Contribution:**

A PAG consisting of wives and daughters (*n* = 4) of people with dementia worked alongside the researchers throughout the intervention development phase of the project. Contributions included (1) sense‐making and interpreting results from a series of intervention development studies, (2) co‐designing the intervention, and (3) disseminating findings, including co‐writing the current paper.

**Abbreviations**
GRIPP2: Guidance for Reporting Involvement of Patients and the Public 2
LI‐BA: Low‐Intensity Behavioural Activation
NIHR: National Institute for Health and Care Research
PAG: Public Advisory Group
SRQR: Standards for Reporting Qualitative Research

## 1 Introduction

Public contribution in research is increasingly prioritised by research institutions, funding bodies, and policymakers internationally [1]. Defined as research carried out *with* or *by* members of the public rather than *to*, *about*, or *for* them [2], public contributors are active research partners with lived experience involved in prioritising, designing, conducting, analysing, and disseminating research [3]. Recognising the importance of the perspectives of those affected by research, while acknowledging that these may differ from those of researchers, is a cornerstone of public contribution [4].

The rationale for involving the public in research rests on several key premises, including the public's right to be involved in research that affects them [5]. Additionally, lived experience and real‐world perspectives can enhance research quality and relevance [5]. Public contribution can also have a positive impact on researchers, including obtaining new perspectives and adding a sense of meaning to their work [6], and on public contributors themselves, including feeling valued and gaining new knowledge [7]. However, negative impacts are also reported, including increased researcher workload and greater resource demands [6], alongside feelings of inadequacy, exhaustion, and distress among public contributors [7].

Whilst there is currently no agreed definition of impact in the wider public contribution literature, the NIHR Public Involvement Impact Working Group define it: “The changes, benefits, and learning gained from the insights and experiences of patients, carers and the public when working in partnership with researchers and others” [8]. However, impact may also be negative [8]. Distinctions are also made between impact on processes (i.e., how activities are conducted) and outcomes (i.e., results of activities). Impact on processes may include level of involvement and influence on decision‐making [9]. Impact on outcomes may include increased knowledge, skills, and empowerment [9]. Impact on outcomes can also result from intervention co‐creation [10], for example, by improving acceptability [11], relevancy [12], and adherence [13]. However, the evidence base for the impact of public contribution has been criticised for being largely anecdotal, potentially stemming from inadequate and inconsistent reporting [3, 14, 15]. There is a need to evaluate and report the impact of public contribution to, for example, justify time and financial resources invested [4] and guide best practices [16, 17]. The experiences of public contributors and researchers are also seldom reported, further limiting our understanding of the impact of public contribution [5].

People with dementia have long been neglected as public contributors [18], potentially due to challenges in maintaining the involvement of people living with a progressive disease [19]. Research involving informal caregivers (caregivers) of people with dementia as public contributors is also scarce [20]. In recent years, however, involving people with dementia as public contributors has slowly increased, and European and international dementia organisations have published best practice position statements concerning public contribution with people with dementia [18]. While the number of published studies reporting public contribution activities in dementia research is increasing, a significant gap still exists in evaluating and reporting such activities [4, 21]. Additionally, most studies evaluating the impact of public contribution in dementia research have been conducted in the United Kingdom [4], and it is important to conduct evaluations in other settings.

### 1.1 The INVOLVERA Project

More than one‐third of people living with dementia experience depression [22]. While evidence‐based psychological interventions can be effective [23], access is limited [24]. This study was conducted in the context of the INVOLVERA project, which aims to develop, test, and evaluate a guided low‐intensity behavioural activation (LI‐BA) intervention for people with dementia and depression in Sweden [25].

LI‐BA is an evidence‐based psychological treatment for depression. It uses a simple, structured, and graded approach to support people with dementia re‐engaging with necessary, routine, and pleasurable activities they have stopped doing, or identifying new activities with similar meaning to them [26]. Within the INVOLVERA intervention, a caregiver supports the person with dementia to use LI‐BA, and the caregiver receives regular guidance from a trained healthcare professional. The intervention is delivered through a printed workbook for people with dementia and a guidebook for caregivers. Separate case stories provide examples of how others have worked with the intervention [25]. The workbook is written in dementia‐friendly language and provides step‐by‐step guidance alongside practical worksheets to aid completion. The guidebook provides caregivers with tools to support the person with dementia to work through the intervention. Illustrations are used to enhance understanding [27].

Working alongside a Public Advisory Group (PAG) comprising caregivers of people with dementia, a series of iterative studies informed adaptation of the intervention originally developed in the United Kingdom [27]. Semi‐structured interviews and focus groups explored needs and preferences, and informed intervention adaptation to enhance cultural appropriateness and relevancy [28]. Barriers and facilitators to intervention uptake have been identified from the perspectives of people with dementia, caregivers, healthcare professionals, and nongovernmental organisations [29, 30].

### 1.2 The INVOLVERA Public Advisory Group

#### 1.2.1 Recruitment

Advertisements to join the PAG were published in Facebook groups for caregivers and people with dementia, with 16 caregiving women and one woman with dementia expressing interest. Due to project delays caused by COVID‐19, recruitment was closed early to establish the PAG quickly rather than continue advertising to recruit people with dementia and caregiving men. Those expressing interest were given information about the PAG and briefly interviewed over the telephone. Questions included sociodemographic background, time caregiving/time since diagnosis, type of dementia, and availability. Striving for diversity in caregiving experiences and sociodemographic characteristics, five public contributors were recruited. Four contributed to public contribution activities, and the fifth member withdrew (reason unknown). One person with dementia was recruited but decided not to join the PAG due to dementia symptoms.

#### 1.2.2 Public Contribution Activities

A study‐focused framework was adopted for involving PAG members [31]. Public contribution activities included (1) sense‐making and interpreting results from studies conducted in the intervention development phase [25, 28, 29, 30], (2) co‐designing the intervention, and (3) disseminating findings.

Public contributors contributed at the ‘involve’ level of the IAP2 Spectrum of Public Participation, working alongside researchers to ensure public concerns and objectives were recognised and addressed [32].

**Figure 1:**
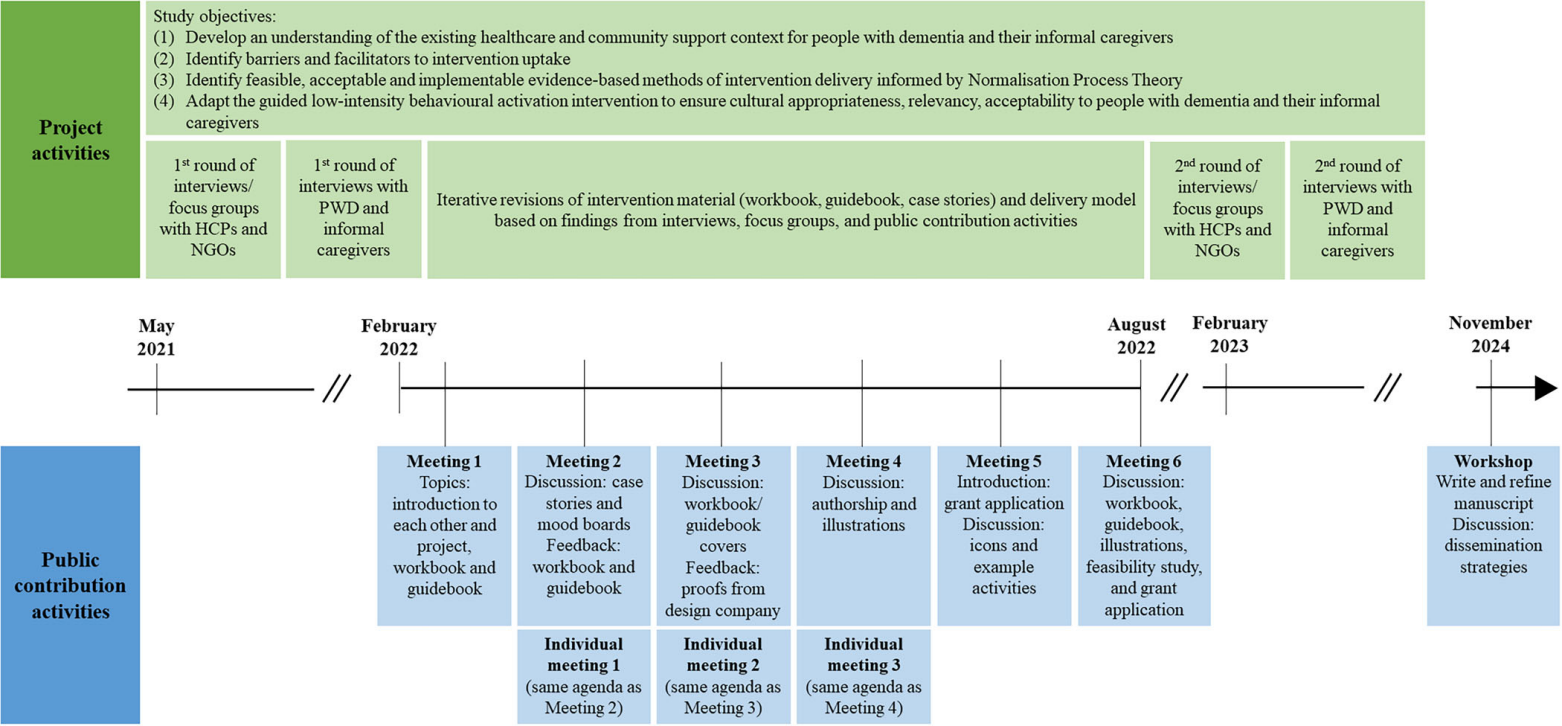
Timeline and overview of project and public contribution activities. HCP = healthcare professional, NGO = nongovernmental organisation, PWD = people with dementia.

Nine PAG meetings were held during the intervention development phase (March–August 2022) with activities and topics shown in Figure 1. Six meetings included two or more PAG members, and three individual meetings were arranged for public contributors unable to attend group meetings. Meetings were held via Zoom and lasted 120 min, with two PhD students facilitating meetings. In November 2024, a 2‐day face‐to‐face workshop was held with public contributors (*n* = 3) and researchers (*n* = 2) to collaboratively write the present paper. The term researcher is used to refer to any research team member, which included research assistants, PhD students, and senior academics.

PAG meetings were coordinated from Uppsala, Sweden. Researchers involved in such activities received training from the principal investigator.

### 1.3 Aim

We explored the experience, process, and impact of involving caregivers of people with dementia as public contributors during the intervention development phase of INVOLVERA.

Objectives explored public contributors and researchers'

1. Experience of public contribution activities.
2. Perceived impact of public contribution activities on the research and themselves.
3. Perspectives on the process of public contribution, including influence on decision‐making.

Furthermore, with respect to public contributors, we explored

4. Ideas for the future development and expansion of the PAG.

## 2 Methods

### 2.1 Researcher Characteristics and Reflexivity

I.Ö.M., a female research assistant (MSc Global Health), conducted the semi‐structured interviews. She was not involved in intervention development or public contribution activities and had no pre‐existing relationship with public contributors, but had a pre‐existing relationship with two researchers interviewed. I.Ö.M. and F.S., a female doctoral student (MSc Public Health), analysed the data. F.S. was involved in intervention development and coordinating public contribution activities and had pre‐existing relationships with public contributors and researchers. A.C.Å., a female professor (Medical Sciences), supervised data collection and data analysis. J.W. a female associate professor (Caring Sciences) and principal investigator, provided peer‐examination. A.C.Å. and J.W. are experienced in qualitative and mixed‐methods research. Additionally, JW has experience working with public contribution in Sweden, Tanzania, and the United Kingdom.

### 2.2 Study Design

This study utilised a convergent parallel mixed‐methods design with qualitative and quantitative data given equal priority [33], and was embedded in the intervention development phase [34] of INVOLVERA [25]. Data was collected using impact logs from PAG meetings and semi‐structured interviews. Impact log data was transformed [35] into quantitative data and integrated with findings from qualitative interviews during interpretation. Reporting follows the GRIPP2 checklist [36] (Supporting Information: File 1) and SRQR checklist [37] (Supporting Information: File 2).

### 2.3 Study Participants

After the intervention development phase, public contributors (*n* = 4) and researchers (*n* = 3) were invited to participate in semi‐structured interviews. All agreed to participate and provided informed consent.

Eligible public contributors were (1) living with dementia or being a caregiver to a person with dementia, (2) able to understand and write in Swedish, and (3) living in Sweden.

Eligible researchers were (1) involved in the development of INVOLVERA, and (2) involved in public contribution activities.

### 2.4 Participant Characteristics

The four public contributor participants were all women residing across Sweden. They were caregivers to people living with dementia; two for their spouses, one for her mother, and one for both her parents and an aunt (all living with dementia). At the time of recruitment, their ages ranged between 44 and 71 years with 5–9 years' experience of caring for someone with dementia. The people they were caring for had diagnoses of Alzheimer's disease (*n* = 4) or vascular dementia (*n* = 2), and their ages ranged from 46 to 88 at the time of dementia symptom onset and need for informal care.

The three researcher participants included two women and one man. At the time of interviews, their ages ranged from 33 to 42 years and their experience of working with public contribution ranged from 2 to 12 years.

### 2.5 Data Collection

#### 2.5.1 Impact Logs

Impact logs were completed by a facilitator after each PAG meeting, recording: date, type of interaction, attendees, discussions, and impact on research, researchers, and public contributors [38]. Impact logs were shared with public contributors to provide feedback on accuracy after meetings.

#### 2.5.2 Semi‐Structured Interviews

Seven interviews (26–53 min) were conducted May–June 2024 via Zoom (*n* = 2), over telephone (*n* = 3), and face‐to‐face (*n* = 2). An interview guide informed by previous research [39] explored public contributors' (1) experience, (2) perceived impact on the research, researchers, and themselves, (3) potential gains in knowledge and skills, (4) perceived impact on decision‐making process, (5) recommendations concerning how to expand the PAG with people with dementia and caregiving men (Supporting Information: File 3). A similar interview guide was used for researchers, without questions regarding PAG expansion (Supporting Information: File 4). All interviews were audio‐recorded.

### 2.6 Data Analysis

#### 2.6.1 Impact Logs

Impact logs were analysed following a three‐step process. First, suggestions made by public contributors were extracted by I.Ö.M. When multiple public contributors made the same suggestion during a PAG meeting, these were merged and recorded as one suggestion. For example, if all four public contributors suggested reducing the amount of workbook text, this was counted as one suggestion. However, if multiple public contributors made different suggestions on the same topic, each was recorded as a separate suggestion. For example, if all four public contributors suggested different workbook titles, this resulted in four suggestions. When public contributors made the same suggestions in different PAG meetings, it was treated as a separate suggestion in each instance, given different versions of the workbook/guidebook were discussed in each meeting. Second, I.Ö.M recorded whether each suggestion had been implemented (yes/no), that is, resulted in changes to the intervention or research project. F.S. reviewed the assessment, with discrepancies discussed with J.W. if needed. Third, the list of suggestions was reviewed by I.Ö.M. for familiarisation, with initial observations regarding potential patterns noted. Subsequently, initial categorisations of suggestions were made. Following discussions with F.S., categorisations were revised by I.Ö.M. and reviewed by F.S., with discrepancies addressed and J.W. consulted if needed. The number of suggestions implemented within each category was recorded and counted by I.Ö.M. and checked by F.S. to transform the impact log data to quantitative data [35], and the percentage of suggestions implemented per category and across all meetings was calculated.

#### 2.6.2 Semi‐Structured Interviews

Interviews were transcribed by an external transcriber. F.S. and I.Ö.M. independently analysed transcripts using qualitative manifest content analysis [40, 41]. An inductive approach was adopted at the manifest level, for example, using a low degree of interpretation (i.e., limiting interpretations beyond what is directly observable in the text) [40, 42]. All analysis activities were recorded in an audit trail. First, F.S. and I.Ö.M. familiarised themselves with data set by independently reading transcripts multiple times [43]. Second, F.S. and I.Ö.M. independently identified meaning units (i.e., words, sentences, or paragraphs with the same central meaning relevant to the research aims), condensed these meaning units, and labelled them with a code [40, 43]. NVivo 15 was used to support the coding process. Third, F.S. and I.Ö.M. individually compared codes for differences and similarities [43], and abstracted subcategories and categories. They subsequently held data analysis workshops, where categorisations were discussed, revised, and reapplied to the data set to ensure credibility [44]. Codes were written on post‐it notes to facilitate discussions. Further data analysis workshops were held with A.C.Å. and subsequently A.C.Å. and J.W. to increase credibility (see Supporting Infiormation: File 5 for photos from data analysis workshops). During workshops, categories and sub‐categories were discussed and revised, to ensure they were externally heterogeneous and internally homogeneous [45]. Next, F.S. wrote dense category descriptions that were sent to I.Ö.M., J.W., and A.C.Å. for feedback. A data analysis workshop was held (F.S., I.Ö.M., J.W., A.C.Å.) to revise categories, sub‐categories, and category descriptions before being sent to the wider team for peer examination. Supporting quotations were selected and translated from Swedish to English by a native Swedish speaker (F.S.), and reviewed by a native English speaker (J.W.). See Table 1 for an example of the analysis process and Supporting Information File 6 for the coding tree.

**Table 1:** Example of the analysis process.

Meaning unit	Condensed meaning unit	Code	Subcategory	Category
Although I have cared for three people with dementia, close ones, I learned quite a lot [from being involved as a public contributor] that I sometimes can remember in everyday life, an understanding or… Well, I can settle into it	Despite caring for three close relatives with dementia, I gained valuable insights that I can adopt in everyday life and find comfort in	Gaining useful techniques for everyday life	Personal reward and skills	Perceived impacts
They treated us with incredible respect, and it was about which names should go where, or whether we would be a research group… a caregiver group or something like that, so you felt very nicely treated	Researchers treated us with respect, including details like how the group should be addressed	Respectful environment	Dynamics	Interactions and facilitators

#### 2.6.3 Mixed‐Methods Integration

A convergent parallel mixed‐methods design was adopted. Quantitative and qualitative findings were integrated during interpretation through a side‐by‐side comparison of the findings in the discussion, enabling interpretation of whether findings aligned, contradicted, or expanded upon one another [33].

### 2.7 Trustworthiness

To increase trustworthiness, we conducted independent coding, adopted a code‐recode procedure, performed peer examinations with experienced researchers, triangulated findings, provided dense descriptions of the methods, reported author reflexivity, and kept an audit trail [44]. Interviews were conducted by a researcher not involved in intervention development or public contribution activities.

## 3 Results

### 3.1 Impact Logs

Public contributors made 158 suggestions during the intervention development phase, with 120 (76%) implemented by the researchers. The categorisation process resulted in seven categories (Table 2). Reasons for not implementing suggestions are provided in Supporting Information: File 7.

**Table 2:** Categorization of public contribution suggestions and number and percentage of those implemented.

Category	Category description	Number of suggestions *N*	Implemented *N* (%)
Intervention case stories	Suggestions related to the inclusion, structure, relatability, and narrative flow and content of case stories	32	28 (88%)
Intervention content	Suggestions related to the clarity and relevance of intervention material, including example enjoyable, routine, and necessary activities	26	23 (88%)
Intervention illustrations	Suggestions related to the style, mood, tone, diversity, and relatability of visual illustrations	43	32 (74%)
Intervention layout	Suggestions related to the design elements, including colour scheme, font, margins, spacing, and use of icons	31	22 (71%)
Intervention language	Suggestions related to word choice, message tone, and overall readability	15	8 (53%)
Intervention text	Suggestions related to sentence and paragraph length, text density, and overall quantity of written content	6	5 (83%)
Future research	Suggestions related to future studies and grant applications	5	2 (40%)
Total		158	120 (76%)

**Figure 2:**
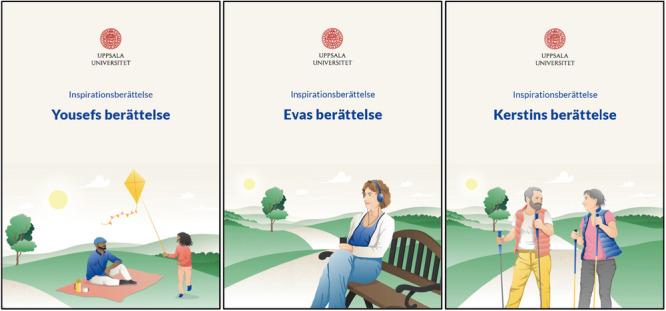
Three separate case stories depicting different life situations. English translation of case stories' titles: Inspirational stories—Yousef's story, Eva's story, and Kerstin's story. Illustration © 2022 ETC Kommunikation.

To illustrate the impact of some implemented suggestions made by the PAG, Figure 2 shows three separate case stories, each depicting different life situations of people with dementia, which aimed to enhance diversity and increase the relevance of INVOLVERA to a broader range of users. Case stories were co‐designed with the PAG to replace the original single case story, which was perceived by study participants in the intervention development phase as depicting stereotypically older adults, negatively impacting intervention relevancy [28].

**Figure 3:**
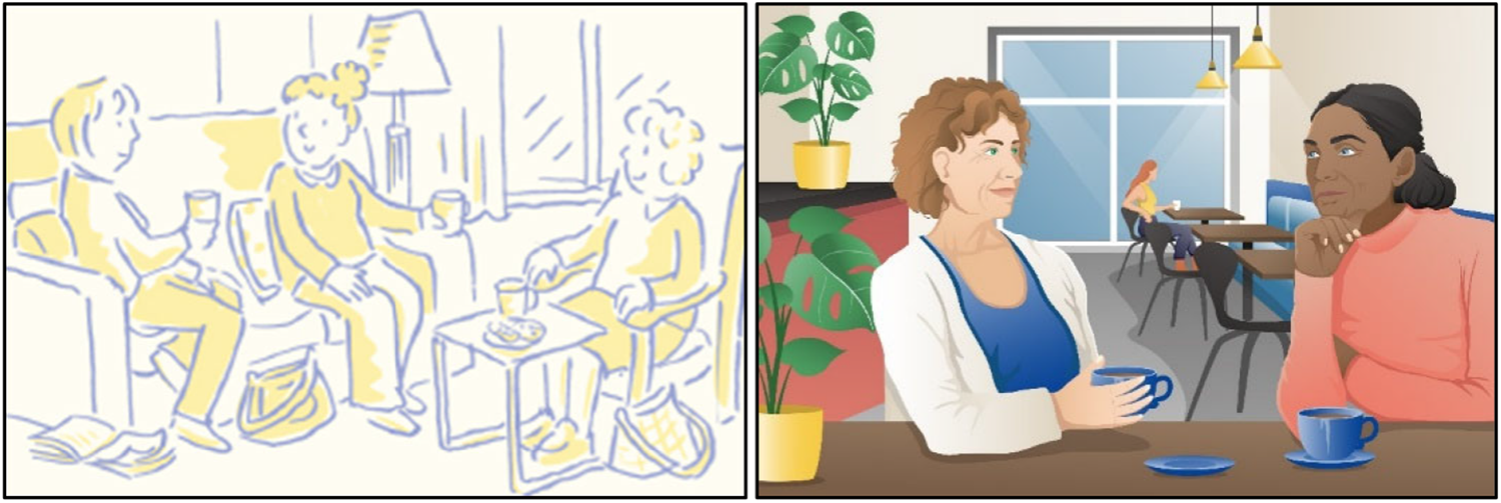
Example of the old and new illustrations. Illustration © 2015 Paul Dowling. Illustration © 2022 ETC Kommunikation.

Original illustrations presented to study participants during the intervention development phase were perceived as outdated and not reflective of broader demographic and societal changes [28]. The PAG helped the researchers to develop ideas for new modern illustrations. An example of one of the original illustrations and new modern illustrations are presented in Figure 3.

### 3.2 Semi‐Structured Interviews

Analysis generated three categories (1) *Perceived impacts*, (2) *Interactions and facilitators*, and (3) *Future challenges and opportunities*, with seven sub‐categories related to the experience, process, and impact of public contribution activities.

#### 3.2.1 Perceived Impacts

This category describes perceived impacts of public contribution activities on the research, intervention, researchers, and public contributors. It includes sub‐categories (1) *Research relevancy and real‐world applicability*, (2) *Intervention adaptation*, and (3) *Personal reward and skills*.

##### 3.2.1.1 Research Relevancy and Real‐World Applicability

Public contributors described their lived experiences as bringing real‐world perspectives to the research:

As a caregiver, living with the disease puts you in the midst of it, maybe allowing you to see challenges differently from someone who doesn't experience it first‐hand… I could provide input on the challenges I experienced. I think that's the largest impact. I live in the real world.

(Public contributor 3)

Researchers also perceived incorporating lived experiences as essential for conducting relevant research that fits real world settings. One researcher expressed public contribution activities as bringing them closer to the real world, gaining confidence that their research was addressing issues that matter to people's everyday lives:

Sometimes in academia, we become so far removed from the real world… I guess sometimes you can maybe think, ‘Am I doing relevant research?’. When you work with public contributors and you're gaining feedback that what you're doing is relevant and important, it feels great.

(Researcher 2)

Researchers reported public contributors had a key role in making sense of and interpreting intervention development study findings. They also supported researchers with ideas for funding applications and identifying research priorities:

One of the meetings was around ideas for a potential grant application and what the focus should be. That was incredibly helpful.

(Researcher 2)

Some potential negative impacts were expressed by researchers, including an increased need for time and funding resources. However, they suggested over the long term, involving public contributors can result in cost savings and enhance relevancy:

On one hand, it's more costly because more people are involved, which adds expenses for travel and working hours. On the other hand, it also reduces waste by making it [research] more relevant, resulting in less funding waste.

(Researcher 3)

##### 3.2.1.2 Intervention Adaptation

Both public contributors and researchers emphasised the importance of public contribution activities during intervention development, expressing it as increasing acceptability, quality, and relevance. Specifically, co‐designing the intervention material was described as improving the presentation structure, layout, readability, and visual content of the workbooks:

We talked about the layout. It was about margins, readability, illustrations, and more graphical examples. I think it turned out well.

(Public contributor 1)

Another public contributor added:

How to make sentences short and simple. So that it wasn't written with too much detail and instead language that was easy to understand.

(Public contributor 4)

Researchers agreed co‐designing the intervention was essential for adapting it to better fit the Swedish context. This included identifying activities reflecting daily life in Sweden, alongside writing case examples representative of Swedish society to increase inclusivity. They also described how public contributors helped improve the language, which was originally translated from English:

Public contributors helped us to identify where the language sounded particularly un‐Swedish, and then how we were able to adapt the language and restructure the sentences.

(Researcher 2)

##### 3.2.1.3 Personal Reward and Skills

Public contributors expressed feeling valued and public contribution activities allowed them to contribute in meaningful ways. They emphasised the importance of helping others experiencing similar challenges:

Living with someone who has Alzheimer's brings so many questions, thoughts, and frustration. It felt very important to be a part of the group to be able to make it a little easier for other caregivers and those living with dementia. That's probably what I felt was most positive and important.

(Public contributor 3)

Both public contributors and researchers described gaining new skills, knowledge, and perspectives. For example, public contributors reported acquiring practical tools from the intervention that could be applied in their everyday lives. They also expressed an increased understanding of dementia, a greater ability to process their own situation, and a sense of relief by sharing experiences with other caregivers. Equally, some voiced wishing they had gained these skills when the person they were caring for was in an earlier dementia stage. Researchers reported increased knowledge in working with and integrating public contribution in research, enhanced communication skills, and increased understanding of the situation of caregivers:

It's deepen my understanding, especially regarding experiences of caregivers and what it's like living with someone with dementia… It's been very helpful since my research mainly concerns caregivers of people with dementia.

(Researcher 3)

No public contributors or researchers expressed negative personal impact.

#### 3.2.2 Interactions and Facilitators

This category describes interactions between public contributors and researchers, and facilitators of these interactions during public contribution activities. It includes sub‐categories (1) *Communication*, and (2) *Dynamics*.

##### 3.2.2.1 Communication

Overall, communication was perceived as good. However, both public contributors and researchers recognised the need to provide more frequent feedback to public contributors to keep them motivated:

We as a lay group don't see how the project is progressing. People might be working very hard behind the scenes… I think the feedback you, as a lay group, get is important. So you keep the group warm.

(Public contributor 2)

The need for more frequent feedback was further illustrated by public contributors agreeing they had been meaningfully involved but expressed uncertainties whether they had been able to contribute to the project:

I'm not sure [if we contributed] … It's like that fumbling white cloud. Could we contribute something? Did we do that?

(Public contributor 1)

Further, uncertainty was expressed concerning involvement in decision‐making. Some public contributors were confident they had been actively involved in decision‐making, others felt their involvement was limited to smaller decisions, and some expressed that they had not been involved in decision‐making but would have liked the opportunity.

Both public contributors and researchers appreciated online meetings, which were perceived as essential for public contributors, enabling involvement despite time constraints and geographical location, with one stating: “*It'd never have worked otherwise”* (Public contributor 1). Involvement was described as flexible and adapted to the needs and availability of public contributors:

I think it was very adapted to them. If someone couldn't attend, we made sure to offer an individual meeting or update them on what they missed, so they wouldn't fall behind or feel left out.

(Researcher 1)

##### 3.2.2.2 Dynamics

Public contributors expressed overall positive group dynamics, highlighting a strong sense of belonging and inclusivity. They emphasised the importance of fostering strong group cohesion and creating an open atmosphere where everyone feels comfortable sharing. Reflecting on positive group dynamics, public contributors described researchers as respectful and: “*Very keen to hear our feedback*” (Public contributor 2).

However, it was acknowledged group dynamics could have been further strengthened through face‐to‐face meetings. While public contributors appreciated the convenience of online meetings, occasional face‐to‐face meetings could have been beneficial:

I think that it would have been good to meet physically. It's a bit more difficult of course, but… I think it would have made quite a difference if you'd been able to meet face‐to‐face.

(Public contributor 4)

Face‐to‐face meetings were perceived as important to facilitate more interactive activities:

It would have been nice if we'd been able to have some face‐to‐face meetings… At least once or twice a year to have some sort of face‐to‐face interactive activity.

(Researcher 2)

While public contributors were positive about recruiting additional members, they expressed concerns about the possible impact on current group dynamics. Specifically, expanding the group size could make interactions and relationships less personal. Both public contributors and researchers voiced dividing a larger PAG into smaller groups in online meetings may facilitate effective interactions:

Maybe divide into two subgroups … perhaps have a meeting with everyone, and then subgroups can continue working on a couple of areas.

(Public contributor 1)

It's good to divide into small groups, especially if the group becomes larger, then you can use breakout rooms on Zoom.

(Researcher 3)

#### 3.2.3 Future Challenges and Opportunities

This category describes public contributors' suggestions for the development and expansion of the PAG, mainly with caregiving men and people with dementia. It includes sub‐categories (1) *Gender differences*, and (2) *Living with dementia*.

##### 3.2.3.1 Gender Differences

Public contributors acknowledged recruiting caregiving men to the PAG as being difficult, with one voicing: *“Men are harder to reach*” (Public contributor 2). One suggestion to facilitate recruitment was to explicitly state caregiving men are being sought in advertisements. Already having a man in the PAG and conducting meetings face‐to‐face were perceived as facilitators. Suggested recruitment sources included caregiver groups, memory clinics, and Facebook groups.

Traditional gender roles and stereotypes, specifically among older adults, were articulated as potentially negatively impacting the involvement of caregiving men:

I believe that for men in this generation [born in the 1940s], the step is much larger… Men of that generation weren't perhaps the ones who most often took care of the children… Suddenly being the one who has to do [the caring], it's a completely different experience.

(Public contributor 2)

Public contributors described men as typically taking on caregiving responsibilities such as providing financial support and practical tasks and thus may not identify as being caregivers. Public contributors also voiced men tend to: *“Just manage the illness at home”* (Public contributor 3) rather than seeking support, which may be a barrier to public contribution. There was one disconfirming case, with one public contributor perceiving no differences in barriers to involving caregiving men and women.

##### 3.2.3.2 Living With Dementia

Challenges recruiting people with dementia to the PAG were recognised. While it was agreed involving people with dementia as public contributors is essential, few recruitment suggestions were made. Recruitment sources mentioned included municipal dementia nurses and Facebook groups:

There's some Facebook groups with those who have been diagnosed and are fairly young… who seem happy to share.

(Public contributor 1)

Once people with dementia have been recruited, public contributors emphasised the importance of making adaptations and providing appropriate support to facilitate active and meaningful involvement, for example, shorter face‐to‐face meetings at an easily accessible location or including a relative as support:

I'm thinking that having like a mentor, or someone who can assist and help, is really key.

(Public contributor 4)

Public contributors articulated the importance of carefully considering dementia‐related symptoms when conducting PAG meetings, including day‐to‐day fluctuations in symptoms, as overlooking them may negatively impact involvement. Other barriers included grieving the new life situation when receiving a dementia diagnosis, insecurity, frustration, and lack of willingness to ‘open up’. Dementia stigma was also perceived as a barrier:

I feel that my mother's generation, the 1940s generation, finds it a bit difficult to talk about topics like this [dementia]. In a way, it's somewhat embarrassing. There's a sense that things should be kept within the walls of the home.

(Public contributor 3)

Conversely, courage, resilience, and being in the early stages of dementia were perceived as facilitators. Public contributors also emphasised the importance of people with dementia recognising and accepting their dementia as a prerequisite for involvement:

They [people with dementia] often don't perceive themselves as ill—they may lack ability to recognise it. However, once they reach a stage of awareness, it [being a public contributor] can also be an opportunity to process [the new life situation]. They can be involved and make a difference.

(Public contributor 4)

Regarding group structure, they suggested to have two separate PAGs—one with caregivers and one with people with dementia. This was considered especially important as a combined group may prevent caregivers from speaking openly. They also expressed separate groups as beneficial for people with dementia, particularly for those in the early stages of dementia:

I think that for those in the early stages of dementia, it might be comforting to have their own group, where they can feel a sense of belonging.

(Public contributor 4)

While the general recommendation was to establish two separate PAGs, an alternative approach would be to have one group but to hold some meetings separately:

I couldn't have done it with my mom, not all meetings. We could've had some meetings [together], but definitely not all. I have noticed with [my mother] that every time we've had meetings, I'd wrap the truth up a lot.

(Public contributor 3)

## 4 Discussion

Currently, there is a significant gap in the literature regarding the evaluation and reporting of public contribution activities in dementia research [4]. Public contributors and researchers' experiences are also seldom reported [5]. We sought to explore the experience, process, and impact of involving caregivers of people with dementia as public contributors during the INVOLVERA intervention development phase. Findings from both impact logs and interviews suggest public contribution activities had a significant impact on the overall research, researchers, and public contributors. Public contribution activities were perceived as positive, with valuable recommendations for future development, improvement, and expansion.

Public contributors and researchers perceived public contribution activities to have a significant impact on intervention development by increasing intervention acceptability and quality. Specifically, the codesign process was perceived as resulting in developing a relevant intervention, with new modernised illustrations better reflecting Swedish society, and the development of three case stories reflective of wider demographic and societal changes. The impact on intervention development was further supported by impact logs results, with the majority (76%) of suggestions made by public contributors implemented by the researchers. These findings are consistent with previous research where public contribution activities are reported to have a positive impact on research [5, 46, 47], including enhancing relevance [5]. However, a scoping review concluded that most public contribution impact relates to increased recruitment and retention in clinical trials, alongside contribution to data collection, analysis, and dissemination [14]. Impact is therefore typically not reported during intervention development. Examining the impact of public contribution during this phase is especially important given intervention adaptation through public contribution can lead to less burden for those delivering and receiving the intervention, enhanced usability, and increased adherence [48].

Public contributors also expressed impacts on themselves, including recognising the importance of their lived experiences in making a meaningful difference and supporting others facing similar situations. This altruistic desire aligns with existing literature [14, 49, 50], highlighting public contributors, including caregivers of people with dementia, voice a desire to share their experiences to support others in their caregiving journeys [50]. Similarly, public contributors have emphasised giving back to the community provides a sense of fulfilment and feeling of worth [20]. Also echoing earlier literature [6, 7, 14, 51], public contributors and researchers in the present study reported gaining new knowledge and skills from public contribution activities.

In contrast to previous research illustrating negative personal impact on public contributors [7, 14, 52, 53], such as frustration, marginalisation, distress, and demanding workload [14], no such impact was reported in the present study. Whilst this may be true, public contributors might have felt reluctant to share negative personal impacts with the research team. Unequal power dynamics is a well‐recognised challenge in public contribution [54]. If public contributors experienced lower authority or influence, such as feeling unheard, or hesitant to speak up, it may have influenced their willingness to share negative experiences. We did not interview the public contributor who withdrew from the PAG who may have reported negative impact. Another explanation may relate to the limitations of using impact logs, which primarily recorded impact on the research. To address this limitation in future studies, impact logs should be used to record wider impacts, for example, by providing more training on how to use them and asking researchers and public contributors to reflect upon and record impacts after meetings [55].

While the experience of public contribution activities was described as positive, public contributors articulated a need for more feedback on their contribution and research progress. Insufficient feedback to public contributors has been reported as a barrier to public contribution [56, 57, 58] and feedback is important for motivation [59]. Public contribution coordinators have an important role as intermediaries between researchers and public contributors by facilitating effective communication [59] and can represent a solution to ensure feedback is routinely provided [59, 60]. Possibly as a result of insufficient feedback, some public contributors expressed uncertainty about whether their contribution had made an impact and were unclear about their role in the decision‐making process. These uncertainties further underscore the importance of feedback.

Public contributors reported positive experiences of online meetings, allowing caregivers from across Sweden to be involved despite limited time due to caring and work responsibilities. This aligns with other research, where public contributors have expressed that online meetings can overcome participation barriers by minimising travel and saving time [61, 62]. However, both public contributors and researchers recognised that face‐to‐face meetings would have facilitated interactive activities and supported group dynamics. Previous studies have identified challenges with online public contribution meetings, including financial constraints for acquiring the necessary equipment, limited digital literacy [63], and reduced communication effectiveness [62]. Specifically, interactions may be diminished or lost, potentially due to reduced non‐verbal communication, resulting in less dynamic discussions [62]. Moving forward, it will be important to explore acceptability and effectiveness of different meeting formats to ensure meaningful and sustained public contribution.

Consistent with prior research [21, 64], we encountered challenges in recruiting people with dementia. People with dementia often face barriers to public contribution, including progressive cognitive difficulties and societal attitudes towards dementia [21]. As noted by public contributors in the present study and in the wider literature, strategies to support people with dementia as public contributors include adopting a flexible approach, planning dementia‐friendly meetings, and providing regular feedback [21]. Public contributors also recognised challenges in recruiting male caregivers. While the reason behind only women expressing interest is unknown, one potential explanation may be traditional gender differences in caregiving experience. Historically, women have predominantly undertaken caregiving responsibilities [65], including caring for people with dementia [66]. Current trends show however, that gender differences in caregiving have narrowed over time [65]. This shift is particularly evident in the Nordic European countries where policies emphasising gender equality and public care services have resulted in more shared caregiving between genders [67]. However, research suggests caregiving men may struggle with the unfamiliar role and changing identity as a caregiver [68]. Informed by the suggestions from public contributors and wider literature, we are currently developing recruitment strategies to involve people with dementia and caregiving men in the PAG for the forthcoming feasibility study.

### 4.1 Strengths and Limitations

We utilised a mixed‐methods approach, allowing us to triangulate quantitative and qualitative data. This provided us with an in‐depth understanding of the experience, process, and impact of public contribution activities [69]. Further, the evidence base for public contribution is currently limited [70] and lacks rigours evaluation of its impact [15], whereas we have systematically examined the impact of public contribution to the intervention development phase.

Some limitations should be considered. We were unsuccessful in including caregiving men and people with dementia, and the PAG included four members whereas guidelines recommend including 8–10 members [71], which may limit the transferability of findings. Public contributors served dual roles as participants (i.e., participating in interviews) and PAG members. While this is not uncommon [55, 72, 73], it may present challenges, including making the roles unclear [74]. Given unclear roles in public contribution can negatively affect involvement [75], it is important to clearly define roles. Similar to public contributors, some researchers also served dual roles. This may present a risk of confirmation and reporting bias. To minimise this risk, researchers with dual roles were not involved in primary data analysis and interviews were conducted by a researcher not involved in public contribution activities. When extracting suggestions from impact logs, each unique suggestion on the same topic was recorded as separate suggestions (e.g., four different suggestions on a workbook title were counted as four different suggestions). Since only one suggestion could be implemented (e.g., selecting one workbook title), the remaining three were not implemented. This approach led to a lower overall percentage of implemented suggestions.

## 5 Conclusion

As captured by impact logs and interviews, public contribution activities had a significant impact on the research and those involved. Public contributors were key in co‐designing the intervention and interpreting findings from the intervention development studies. Notably, researchers implemented 76% of suggestions made by public contributors. Both public contributors and researchers reported overall positive experiences. Nevertheless, some suggestions for improvement were identified (1) arranging occasional face‐to‐face meetings to facilitate relationship‐building and support more interactive activities, (2) providing more regular feedback to public contributors to support motivation and sustain involvement, and (3) adopting effective recruitment strategies to involve caregiving men and people with dementia to ensure a broader range of perspectives.

## Author Contributions

**Frida Svedin:** methodology, validation, formal analysis, investigation, resources, data curation, writing – original draft, visualisation, project administration, funding acquisition. **Ida Österman Menander:** formal analysis, investigation, data curation, writing – original draft, visualisation. **Oscar Blomberg:** writing – review and editing, project administration. **Anders Brantnell:** writing – review and editing. **Paul Farrand:** writing – review and editing. **Theresia Lückner:** writing – review and editing. **Kristina Sundelin:** writing – review and editing. **Joan Turney:** Writing – review and editing. **Anna Cristina Åberg:** formal analysis, writing – review and editing, supervision. **Joanne Woodford:** conceptualisation, methodology, writing – original draft, supervision, project administration, funding acquisition.

## Disclosure

During the preparation of this manuscript the principal author used ChatGPT to improve writing style, and check for grammar and spelling. After using the tool, the authors reviewed and edited the content as needed and take full responsibility for the content of this manuscript.

## Ethics Statement

The study was ethically approved by the Swedish Ethical Review Authority [Dnr: 2020‐05542, 2021‐00925, 2021‐05413‐02, and 2024‐00323‐02]. Informed consent was obtained from all participants. All procedures were conducted in accordance with the Declaration of Helsinki. Confidentiality is an important ethical issue when conducting research on PAG members and researchers as the risk of loss of anonymity is high as their names have been, or will be, published in other studies related to the present research project.

## Conflicts of Interest

The authors declare no conflicts of interest.

## Supporting information


**Supporting file 1:** Guidance for Reporting Involvement of Patients and the Public 2 (GRIPP2) checklist.

**Supporting file 2:** Standards for Reporting Qualitative Research (SRQR) checklist.

**Supporting file 3:** Interview guide for public contributors.

**Supporting file 4:** Interview guide for researchers.

**Supporting file 5:** Photos from data analysis workshops.

**Supporting file 6:** Coding tree.

**Supporting file 7:** List of suggestions not implemented and reasons behind decisions.

## Data Availability

Data sets generated and/or analysed during the current study are not publicly available due to privacy or ethical restrictions but are available from the corresponding author on reasonable request.
