# Supplementary material for: Experience, Process, and Impact of Involving Informal Caregivers of People With Dementia as Public Contributors to Inform the Development of a Complex Intervention: A Mixed‐Methods Study
Source: Health Expect. 2025 Aug 17;28(4):e70382. doi: 10.1111/hex.70382 (PMC12358738; doi:10.1111/hex.70382)
Supplement: Supplementary file 3 — Supporting file 3: Interview guide for public contributors. [file HEX-28-e70382-s004.docx]

**Interview guide**

**Public contributors**

**General experiences**

1. To start with, can you share your thoughts on how you experienced being a member of the Public Advisory Group in the INVOVLERA project?
   1. What went well?
   2. What could be improved?
2. Do you have any thoughts on why it was important to involve the Public Advisory Group in the INVOLVERA project?
3. What challenges or difficulties do you feel you have encountered as a member of the Public Advisory Group?
   1. Thinking instead about other informal caregivers of people with dementia, what might prevent them from becoming members of a Public Advisory Group?
4. What are your thoughts on what has made it easier for you to be involved in the Public Advisory Group?
   1. Thinking instead about other informal caregivers of people with dementia, what could make it easier for them to be members of a Public Advisory Group?
      Is there anything else that could be done to support other informal caregivers of people with dementia be part of a Public Advisory Group?

**Impact**

1. Considering that you have been contributing to a research project, do you have any thoughts about the impact your contribution has had on the research?
2. Looking instead at the researchers, what impact do you think your contribution had on them?
3. Turning to yourself, what impact did your contribution have on you personally?
4. How do you think the Public Advisory Group influenced the design of the INVOLVERA intervention?

**Skills and knowledge**

1. When it comes to skills and knowledge, do you feel that you have gained any new skills after being a member of the Public Advisory Group? Or have you further developed any skills you already had?
   1. If yes, which ones? Could you elaborate a bit on the skills you have gained or developed?
2. Focusing on research, what are your thoughts on what you have learned about research after being a member of the Public Advisory Group?

**Decision-making**

1. In research, many different decisions are made. Do you feel that you were involved in that process?
   1. If yes, in what way were you involved in the process? How do you think your involvement influenced the process?
2. Overall, what are your thoughts on how your opinions may have influenced the final result of the research?

**Expanding the group**

We think it has been very valuable and important that you and the others in the Public Advisory Group have been involved, and we would like to continue having a Public Advisory Group. But as you may know, the current group only consists of caregiving women, and we are considering how we could expand the group to also include people with dementia.

1. Do you have any thoughts on how we can involve people with dementia to be part of the Public Advisory Group?
   1. What do you think could make it difficult for people with dementia to be involved in a Public Advisory Group?
   2. What do you think could make it easier for people with dementia to be involved in a Public Advisory Group?

If we continue exploring expanding the group, but this time think about caregiving men who are also not currently represented in the group.

1. Do you have any thoughts on how we can involve caregiving men to be part of the Public Advisory Group?
   1. What do you think could make it difficult for caregiving men to be involved in a Public Advisory Group?
   2. What do you think could make it easier for caregiving men to be involved in a Public Advisory Group?
2. If we managed to to recruit more members to the current Public Advisory Group. How would you feel about that?

[Prompts: If it becomes a larger group, could meetings still be conducted in the same way as now? Or would the meetings need to be held differently? How would it work to have a larger group on Zoom? Do you think informal caregivers and people with dementia should be in the same group, or should they be in separate groups?]

1. Would you recommend other informal caregivers to join a Public Advisory Group in a research project?
   1. If yes, could you share your thoughts on that?
   2. If no, could you share your thoughts on that?
2. We’re almost at the end of the interview.
   [Brief summary].
   First, I would like to thank you for your participation. Then I also would like to ask if there is anything you would like to add that you have not had the chance to say during the interview?
