# Supplementary material for: Experience, Process, and Impact of Involving Informal Caregivers of People With Dementia as Public Contributors to Inform the Development of a Complex Intervention: A Mixed‐Methods Study
Source: Health Expect. 2025 Aug 17;28(4):e70382. doi: 10.1111/hex.70382 (PMC12358738; doi:10.1111/hex.70382)
Supplement: Supplementary file 4 — Supporting file 4: Interview guide for researchers. [file HEX-28-e70382-s001.docx]

**Interview guide**

**Researchers**

**General experiences**

1. To start with, can you share your thoughts on how you experienced working with the Public Advisory Group in the INVOVLERA project?
   1. What are your thoughts on what went well?
   2. What are your thoughts on what could be improved?
2. Why do you think it was important to involve a Public Advisory Group in the INVOLVERA project?
3. Thinking about yourself, what impact might the Public Advisory Group have had on your confidence conducting activities related to public contribution in research?

**Impact**

1. What impact has the Public Advisory Group’s involvement had on the research?
2. Do you think the Public Advisory Group’s involvement have impacted the research group?
   1. If yes, how?
   2. If no, no impact at all?
3. Looking instead at the members of the Public Advisory Group, what impact do you think their involvement had on themselves?
4. Earlier, we talked about what impact might the Public Advisory Group have had on your confidence conducting activities related to public contribution in research, if we disregard confidence, do you think the Public Advisory Group has had any impact on you overall?
   1. If yes, how?
   2. If no, no impact at all?
5. How did the Public Advisory Group impact the design of the INVOLVERA intervention?

**Skills and knowledge**

1. What are your thoughts on the skills and knowledge you have gained when working with the Public Advisory Group? Or have you perhaps further developed skills you already had?

**Decision-making**

1. Can you explain how the Public Advisory Group was involved in decision-making? Did their involvement influence the final result?
   1. If yes, could you share a bit on how their involvement impacted the results?
2. We’re almost at the end of the interview.
   [Brief summary].
   First, I would like to thank you for your participation. Then I also would like to ask if there is anything you would like to add that you have not had the chance to say during the interview?
