# Supplementary figures and images for: Experience, Process, and Impact of Involving Informal Caregivers of People With Dementia as Public Contributors to Inform the Development of a Complex Intervention: A Mixed‐Methods Study
Source: Health Expect. 2025 Aug 17;28(4):e70382. doi: 10.1111/hex.70382 (PMC12358738; doi:10.1111/hex.70382)

**Photos from data analysis workshops**


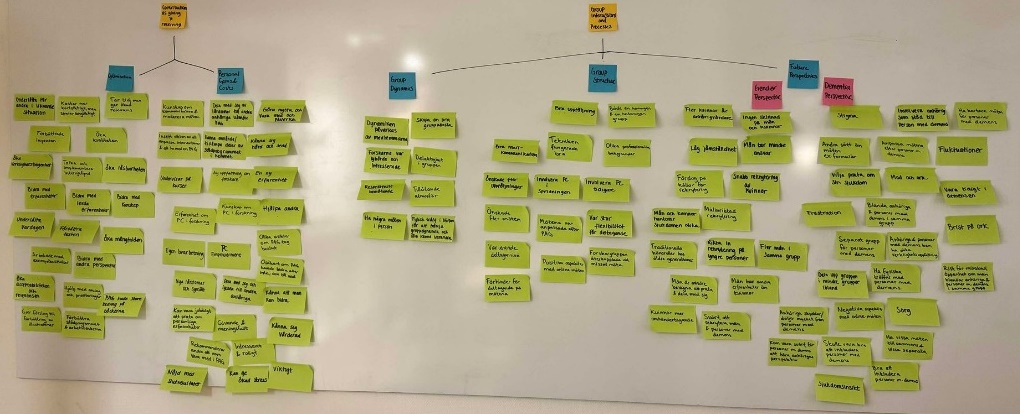

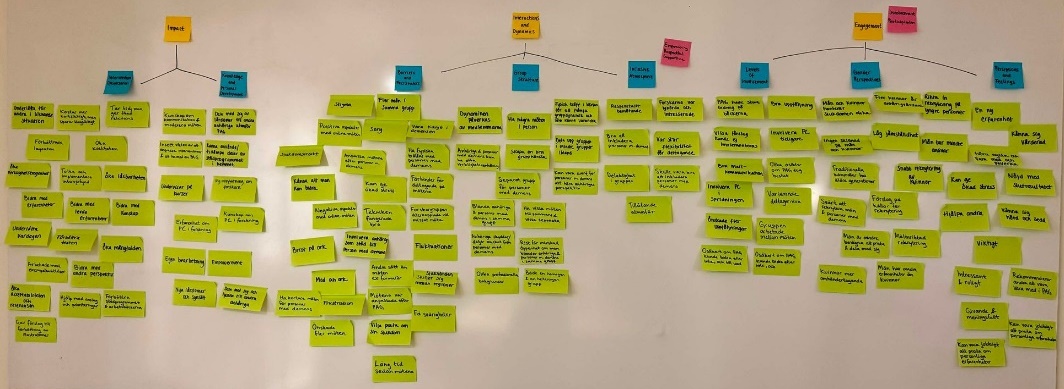
Workshop 1 Workshop 2


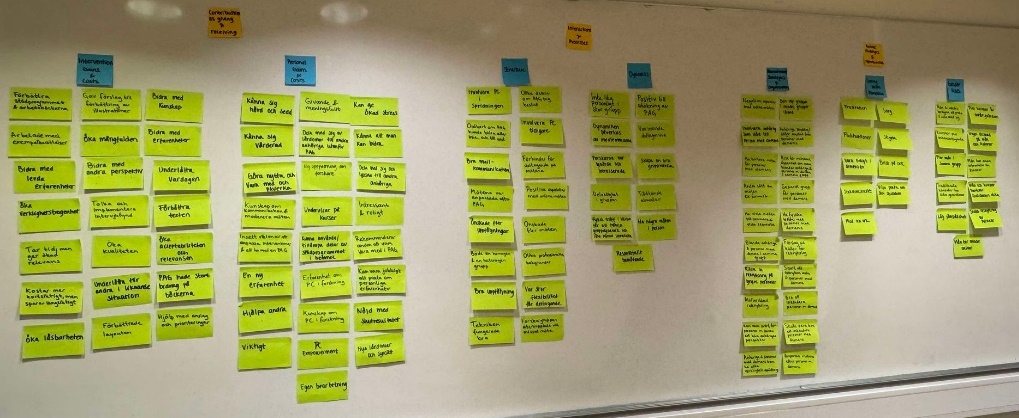

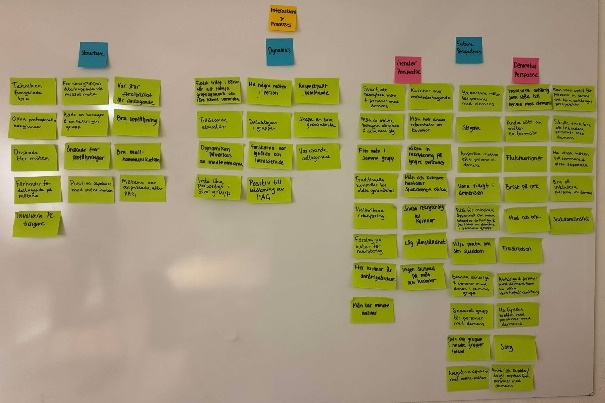

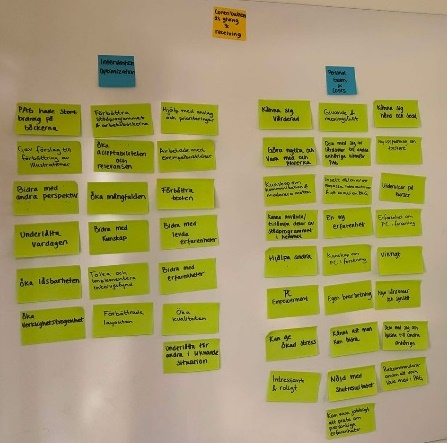
Workshop 3 Workshop 4

Supplement: Supplementary file 5 — Supporting file 5: Photos from data analysis workshops. [file HEX-28-e70382-s003.docx]
