# Supplementary material for: Experience, Process, and Impact of Involving Informal Caregivers of People With Dementia as Public Contributors to Inform the Development of a Complex Intervention: A Mixed‐Methods Study
Source: Health Expect. 2025 Aug 17;28(4):e70382. doi: 10.1111/hex.70382 (PMC12358738; doi:10.1111/hex.70382)
Supplement: Supplementary file 6 — Supporting file 6: Coding tree. [file HEX-28-e70382-s005.pdf]

Coding tree

Supplementary file 6

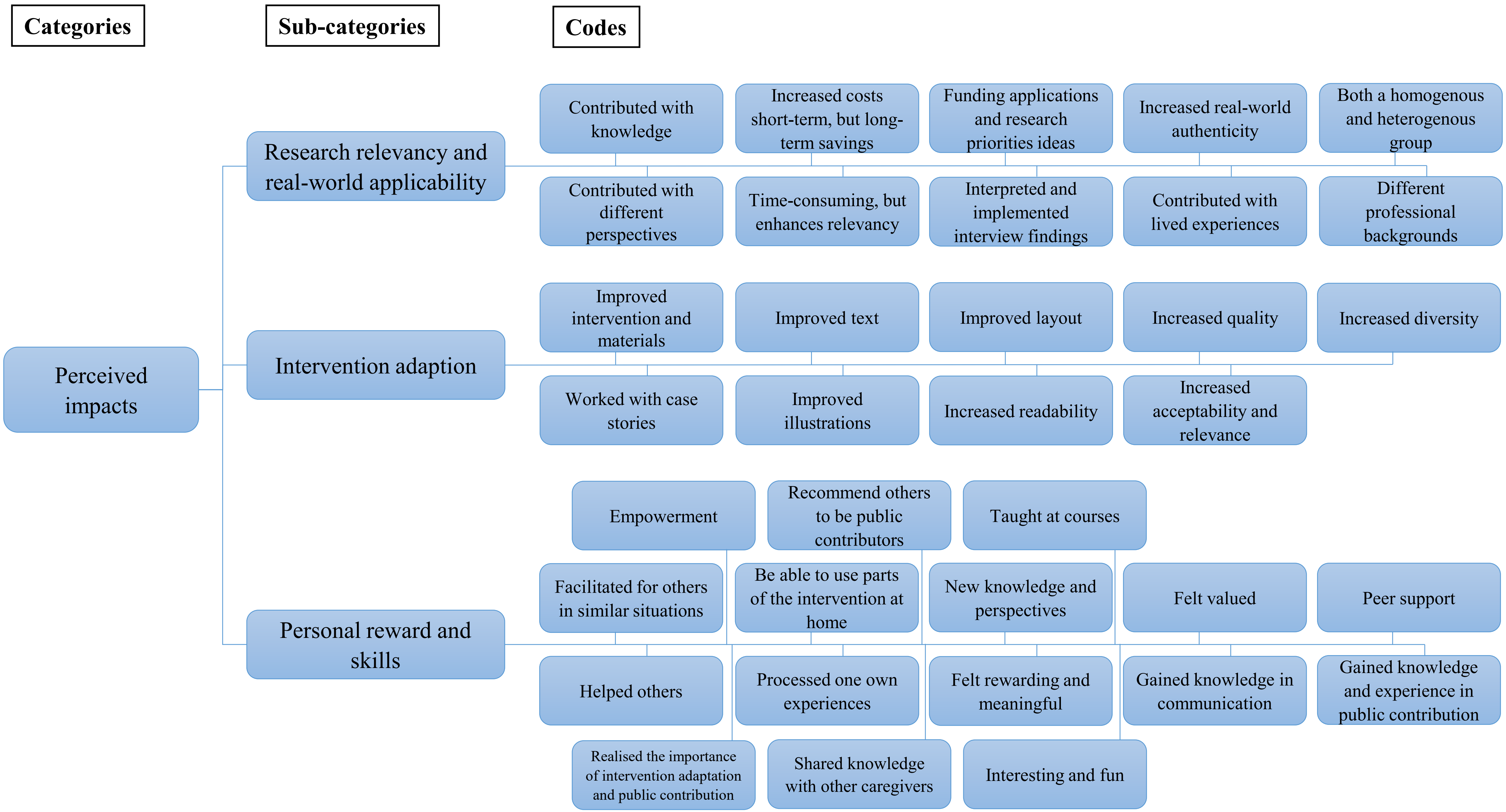

Note. All codes are on the same level.

Coding tree

Supplementary file 6

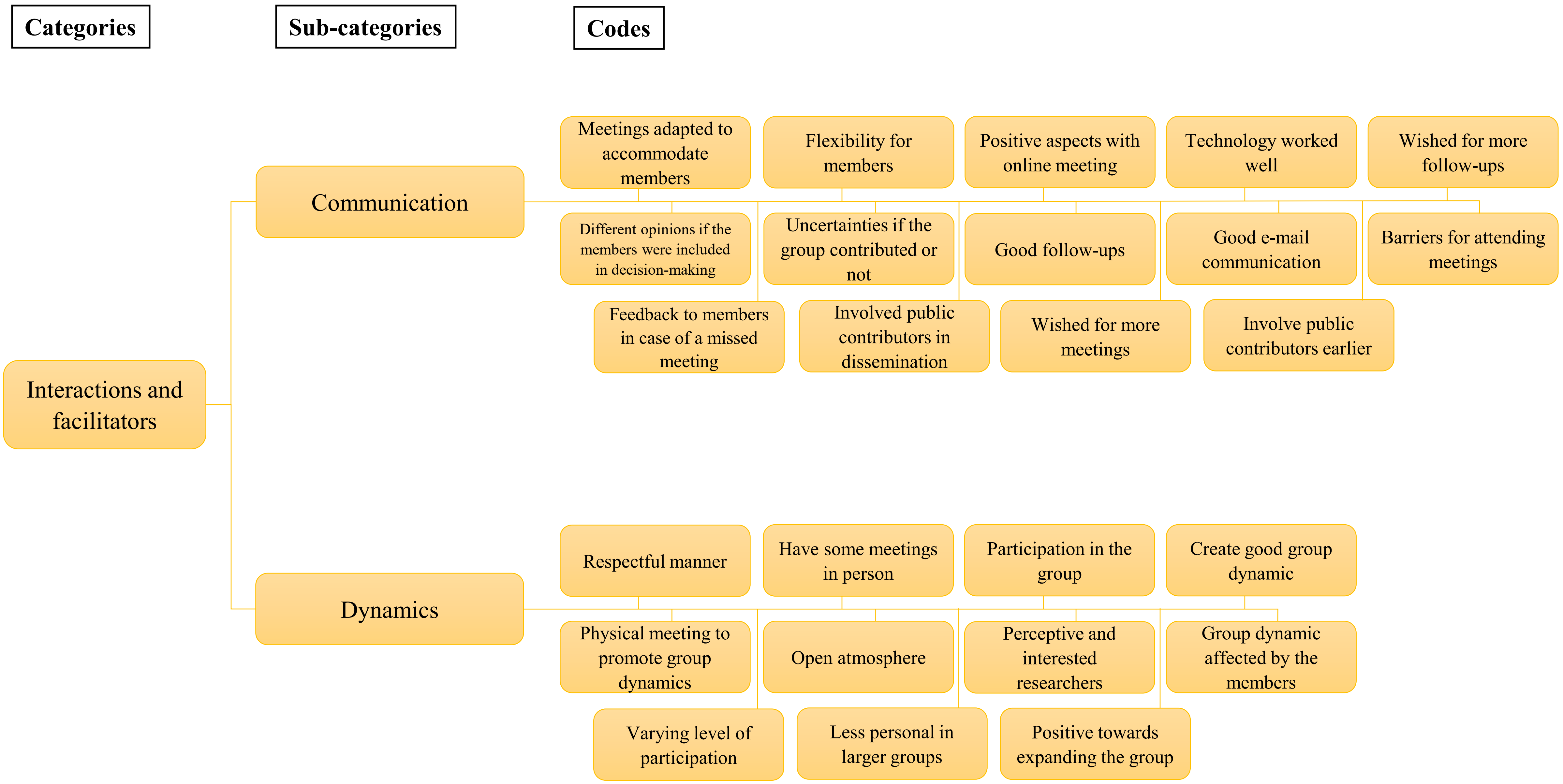

Note. All codes are on the same level.

Coding tree

Supplementary file 6

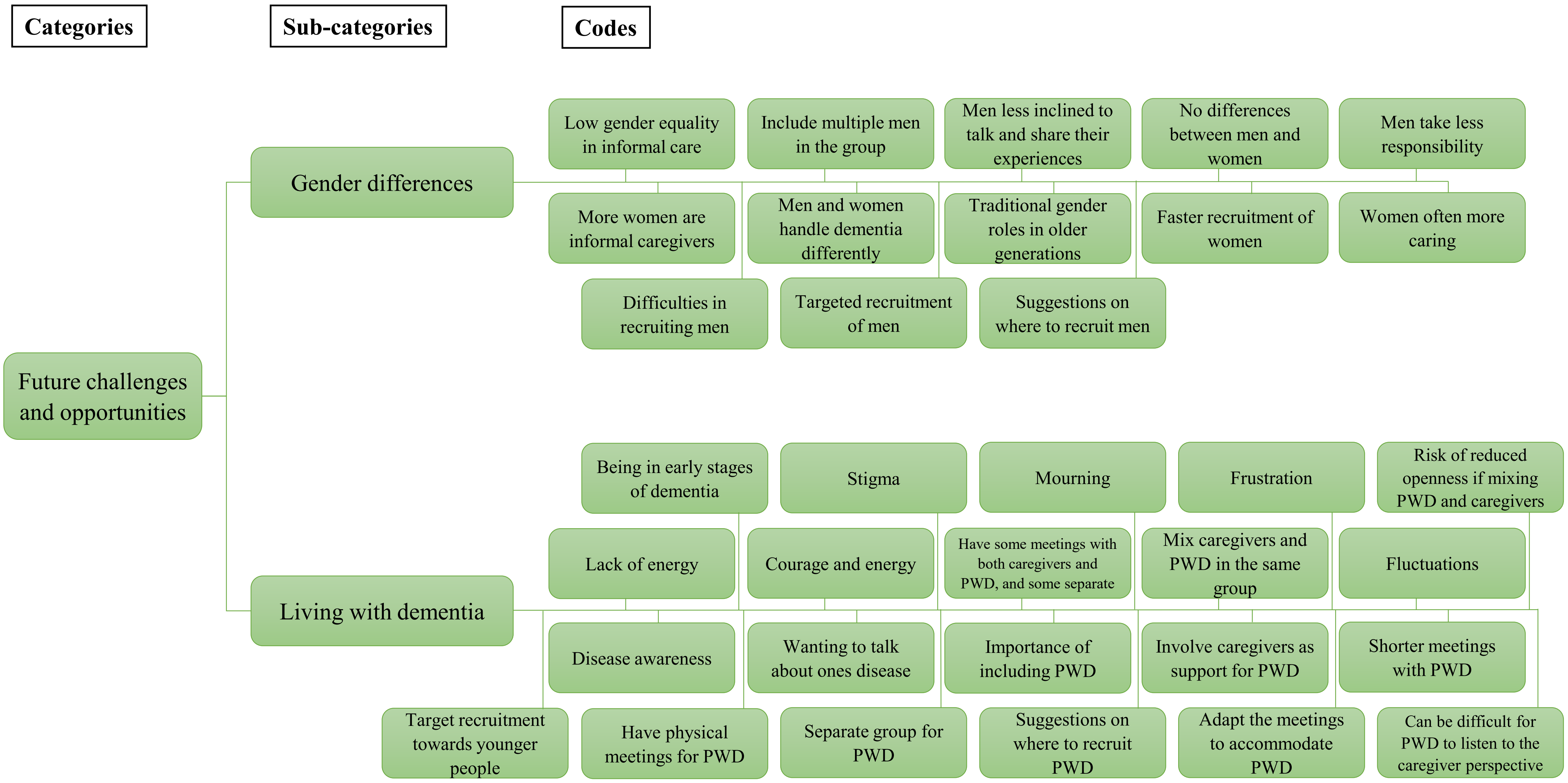

Note. All codes are on the same level. People With Dementia = PWD
