# Supplementary material for: Experience, Process, and Impact of Involving Informal Caregivers of People With Dementia as Public Contributors to Inform the Development of a Complex Intervention: A Mixed‐Methods Study
Source: Health Expect. 2025 Aug 17;28(4):e70382. doi: 10.1111/hex.70382 (PMC12358738; doi:10.1111/hex.70382)
Supplement: Supplementary file 7 — Supporting file 7: List of suggestions not implemented and reasons behind decisions. [file HEX-28-e70382-s006.docx]

**Table 1.** Public contribution suggestions not implemented and reason behind decision.

|  | **Suggestion** | **Reason for not implementing** |
| --- | --- | --- |
| **Meeting 1** | Use more of the red colour in the intervention material | Red can be perceived as negative and related to fear associated emotions. |
|  | Replace illustrations with real pictures in the intervention material | According to the Dementia Engagement and Empowerment Project (DEEP) guidelines, it is important for people with dementia to ensure illustrations/pictures closely align with the text they illustrate to maintain coherence and clarity. Using real photos that closely aligned with the text would require hiring actors and staging scenes, which would be complex and resource intensive. |
|  | Include more pictures of nature in the intervention material | Designing more illustrations of nature was not possible due to financial considerations. |
|  | Define terms frequently used in the intervention material (e.g., worksheet, diary) | The intervention guide will provide a comprehensive introduction of all key components of the intervention material, making definitions unnecessary. Also, another recommendation from public contributors was to reduce text. |
|  | Include space for people with dementia to paste in own pictures in the intervention material | Findings from interviews and focus groups with stakeholders suggested incorporating space for personal pictures might be perceived as childish. |
|  | Include other cultures in the intervention material | The intervention material was adapted to improve representation; however, constraints related to the length of the intervention materials and available resources limited the ability to further expand representation or include other cultures. Future research will explore further cultural adaptations for different cultural groups. |
|  | Make paragraphs shorter in the intervention material | Each paragraph is communicating a certain message to the reader, and splitting might lead to confusion. |
|  | Write the workbook and guidebook in first person | The workbook/guidebook should ‘talk’ to the reader (and deliver the intervention). Using a first-person narrative could make it difficult for readers to relate to the content if it does not apply to their specific situation, potentially leading to lack of recognition/relevance of the intervention material. |
|  | Place case story in the end of the workbook | Three separate case story books were developed to minimize confusion and provide people with dementia and informal caregivers with the option to read case stories if interested/finding it relevant. |
|  | Include multiple case stories in the end of the workbook | See above |
|  | Keep the illustration of the woman in the kitchen cooking food (one of the old illustrations used in the English workbook) | New modern illustrations were designed by a professional design company, replacing all old illustrations. |
|  | Use the term “*tutor*” in guidebook title | The term “*guide*” was used instead as it better reflects the role of the informal caregiver. |
|  | Use the workbook and guidebook title “*get more out of life*” | Another title recommendation from public contributors was chosen. |
|  | Use the workbook and guidebook title “*good life with memory difficulties*” | Another title recommendation from public contributors was chosen. |
|  | Use the workbook and guidebook title “*life quality* *with memory difficulties*” | Another title recommendation from public contributors was chosen. |
| **Meeting 2** | Use a real picture on the workbook and guidebook frontpage | According to the Dementia Engagement and Empowerment Project (DEEP) guidelines, it is important for people with dementia to ensure illustrations/pictures closely align with the text they illustrate to maintain coherence and clarity. Using real photos that closely aligned with the text would require hiring actors and staging scenes, which would be complex and resource intensive. |
|  | Allow people with dementia and informal caregivers to paste their own picture on the workbook and guidebook frontpage | A consistent frontpage design would be more professional and can also help with branding, i.e., facilitate intervention users recognise the intervention, which may also help instil trust. |
|  | Provide options to choose between different frontpages of the workbook and guidebook | Have multiple frontpage options could create confusion and reduce the recognisability of the intervention. |
|  | Use the term “*tutor book*” in the guidebook title | The term “*guidebook*” was used instead as it better reflects the role of the informal caregiver. |
|  | Use the workbook and guidebook title “*live well with memory difficulties*” | Another title recommendation from public contributors was chosen. |
|  | Describe more work tasks of dementia nurses in the intervention material | Another recommendation from public contributors was to reduce text and describing work tasks of dementia nurses was judged less important in the context of this intervention. |
|  | Include some longer and some shorter case stories | Not possible to shorten case studies as a certain amount evidence-based content must be included. |
| **Meeting 3** | Use slightly larger font size in the workbook | Font size was already in accordance with the Dementia Engagement and Empowerment Project (DEEP) guidelines. |
|  | Increase space between the text and the lines on page 11 in the workbook and page 13 in the guidebook | In collaboration with the design company, this specific spacing was decided not possible. However, the overall design of the intervention material was made more spacious to enhance readability. |
|  | Use gears instead of arrows in the illustration depicting a well-being cycle | Arrows were chosen as they provide a simpler and clearer way to illustrate the well-being cycle. |
|  | Reduce the number of people included in the park illustration on page 15 in the workbook | The number of people in the illustration was intentionally maintained to ensure representation of diverse people with dementia and informal caregivers to promote inclusivity. |
|  | Keep the triangle in the layout on page 16 in the workbook | In collaboration with the design company, it was decided to remove the triangle to ensure consistency throughout the layout and design. |
|  | Place the Uppsala University logotype on the backpages of the intervention material | The Uppsala University logotype was placed on the frontpages to ensure visibility and strengthen the association with the university. |
|  | Use the picture of the boat (one of the suggestions of potential pictures to be used) on the frontpage of the workbook | Illustrations were retained for consistency with the workbook material. |
| **Meeting 4** | Adjust the illustration (an illustration of multiple people in an urban setting) so that the child looks out the window at the adults washing the car on the street, rather than engaging in the family dinner | This illustration was designed to depict different family and life situations rather than a single, unified scene. The child is supposed to look at her father with dementia and engage in their family dinner. |
|  | Expand example activities (i.e., examples of activities to enhance understanding or give inspiration) that are included in the intervention material | Including more examples of activities would be too directive and may also result in users not engaging with the intervention, e.g., if they do not identify with activities listed. The intervention is designed to encourage people with dementia to identify activities they personally find of meaning and value. |
| **Meeting 5** | Use icons in the workbook and guidebook | Designing icons were not possible due to financial considerations. |
|  | Make icons blend in in the workbook and guidebook | Designing icons were not possible due to financial considerations. |
|  | Use icons of a light bulb and pen in the workbook and guidebook | Designing icons were not possible due to financial considerations. |
|  | Use maximum 2-3 icons in the workbook and guidebook | Designing icons were not possible due to financial considerations. |
|  | Focus on both people with dementia and informal caregivers in the new grant application | Grant application was not submitted due to time constrains. |
| **Meeting 6** | New grant application should focus on improving the communication strategies used during the COVID-19 pandemic, including interpersonal communication between people with dementia and informal caregivers, as well as broader public communication efforts at societal level | Grant application was not submitted due to time constrains. |
|  | New grant application should focus on the ethical dilemmas related to visiting people with dementia who might be nearing end of life during pandemics | Grant application was not submitted due to time constrains. |
